# Trisomy silencing by XIST normalizes Down syndrome cell pathogenesis demonstrated for hematopoietic defects in vitro

**DOI:** 10.1038/s41467-018-07630-y

**Published:** 2018-12-05

**Authors:** Jen-Chieh Chiang, Jun Jiang, Peter E. Newburger, Jeanne B. Lawrence

**Affiliations:** 1 Department of Neurology and Pediatrics, University of Massachusetts Medical School, 55 Lake Avenue North, Worcester, MA 01655 USA; 2 Departments of Pediatrics and Cancer Biology, University of Massachusetts Medical School, 55 Lake Avenue North, Worcester, MA 01655 USA

## Abstract

We previously demonstrated that an integrated *XIST* transgene can broadly repress one chromosome 21 in Down syndrome (DS) pluripotent cells. Here we address whether trisomy-silencing can normalize cell function and development sufficiently to correct cell pathogenesis, tested in an in vitro model of human fetal hematopoiesis, for which DS cellular phenotypes are best known. *XIST* induction in four transgenic clones reproducibly corrected over-production of megakaryocytes and erythrocytes, key to DS myeloproliferative disorder and leukemia. A contrasting increase in neural stem and iPS cells shows cell-type specificity, supporting this approach successfully rebalances the hematopoietic developmental program. Given this, we next used this system to extend knowledge of hematopoietic pathogenesis on multiple points. Results demonstrate trisomy 21 expression promotes over-production of CD43^+^ but not earlier CD34^+^/CD43^−^progenitors and indicates this is associated with increased IGF signaling. This study demonstrates proof-of-principle for this epigenetic-based strategy to investigate, and potentially mitigate, DS developmental pathologies.

## Abstract (web summary)

Individuals with Down Syndrome have hematopoietic abnormalities including high risk of leukaemia. Here the authors show that transcriptional silencing of one chromosome 21 by *XIST* effectively corrects cell function and development to prevent excessive production of megakaryocytes and erythroids, shown during hematopoietic differentiation of human iPSCs in culture.

## Introduction

Down syndrome (DS), caused by trisomy 21, occurs in about every 750 births in the United States and impacts millions worldwide, with enormous medical and social costs. Children with DS are typically sociable, valued members of families, challenged with mild to moderate cognitive disability that often progresses in adulthood, as well as higher risks of several medical challenges; these include congenital heart disease, high susceptibility to viruses and immune defects, metabolic changes, early-onset Alzheimer disease, and hematopoietic abnormalities, including leukemia. Biomedical research to develop therapies for DS has lagged that of rare monogenic disorders, such that specific DS cell pathologies are mostly unknown, nor is it known how many of ~300 genes on chromosome 21 have any phenotypic effect when present in three copies. Inbred mouse models of DS have been valuable and a number of candidate genes implicated^1,2^, but, with the exception of the known role of *APP* in Alzheimer disease, chromosome 21 genes that underlie major DS phenotypes have yet to be determined. In fact, alternative concepts of DS hold that much of the syndrome is not due to specific chromosome 21 genes but to the physical presence of an extra chromosome causing general stress or cell-cycle defects that impact cell function and vitality^3^. Although aneuploidy is common in cancer, studies in yeast and normal mouse cells show that normally an additional copy of any chromosome causes a proliferative disadvantage, likely due to the proteomic stress caused by collective low-level over-expression of many genes, rather than a few specific dosage-sensitive genes^4,5^.

We previously demonstrated that chromosome 21 over-expression can be countered by epigenetic repression following site-directed insertion of a single gene, *XIST*, into one chromosome 21, shown in DS induced pluripotent stem cells (iPSCs)^6^. The X-linked *XIST* gene naturally controls X-chromosome inactivation in human female cells, producing a long non-coding RNA that coats the X chromosome *in* *cis* to induce a series of chromatin modifications that stably silence transcription across that X chromosome^7,8^. Insertion of *XIST* into a trisomic autosome allowed Jiang et al.^6^ to demonstrate that in absence of selection against silencing (as occurs for a disomic autosome), *XIST* had a remarkably comprehensive capacity to repress genes across the autosome. This prior study focused on demonstrating transcriptional repression throughout the autosome; this was shown in undifferentiated iPSCs using several methods, including allele-specific gene expression, CpG promoter methylation, heterochromatin hallmarks, and genome expression profiling, which showed total chromosome 21 transcriptional output reduced to near normal disomic levels^6^.

Here we address the critical next question: can trisomy silencing (epigenetic repression of one extra chromosome) effectively normalize or mitigate defects in cell function and pathogenesis, which underlie DS phenotypes? A priori, it cannot be assumed that *XIST*-mediated transcriptional repression would be sufficiently robust to correct cell pathogenesis, even in cells that still must carry the physical presence of the extra chromosome 21. Hence, direct determination of this is important for any future prospect of chromosome therapy, or for the utility of this experimental approach (inducible trisomy silencing) to investigate trisomy 21 effects on developmental pathogenesis, for any cell type. We test this in an in vitro model of human fetal hematopoiesis, for which DS cellular phenotypes are best characterized^9–12^. Hematopoietic abnormalities are important clinically, most acutely for 20–30% of infants that develop transient myeloproliferative disorder (TMD), often a precursor to leukemia. Trisomy 21 confers approximately 500-fold greater incidence of acute megakaryocytic leukemia (AMKL) and a ~20-fold greater risk for acute lymphoblastic leukemia (ALL). In addition, less severe hematopoietic abnormalities are present in most individuals with DS, including immune system defects and high susceptibility to viral infections, and chronic inflammation that may contribute to cognitive impairment and decline^13–18^.

Finally, because DS hematopoiesis involves overproduction of certain cell types, comparing the effects of trisomy silencing in hematopoietic cells with neural progenitor cells allows us to address an important question: whether trisomy silencing may enhance cell proliferation/fitness in a nonspecific manner due to relief of aneuploidy stress, not necessarily by correcting specific defects in a developmental program. A priori, silencing trisomy 21 in the hematopoietic system might actually increase the over-proliferation of the trisomic hematopoietic cell types. Alternatively, trisomy silencing may normalize overproduction of these blood cell types, indicating successful correction of a specific defect by normalizing the hematopoietic developmental program.

Several aspects of DS hematopathogenesis have been shown recapitulated in studies comparing human trisomic and disomic iPSCs, providing a benchmark for us to address whether *XIST*-induced trisomy silencing can largely normalize DS-related hematopoietic phenotypes^12,19,20^. In addition, the inducible system used here avoids variation inherent in comparing distinct cell clones, allowing us to not only corroborate but extend knowledge of the specific steps in hematopoiesis affected by trisomy 21. Results show that trisomy 21 over-expression promotes excess CD43^+^ progenitors, but not the earlier CD34^+^ hemogenic endothelium (HE) population. Notably, we use this approach to investigate the as yet unconfirmed hypothesis that overactive insulin-like growth factor (IGF) signaling is present and important in trisomy 21-associated myeloid disorders^21,22^. Results further address whether trisomy 21 alone impacts IGF signaling, prior to the *GATA1s* mutation, which is consistently present in TMD and AMKL leukemic blasts^23,24^. Trisomy 21 itself causes excessive production of erythroid and megakaryocytic cells, which can be observed in fetal liver, or in iPSC-derived hematopoietic cells (without *GATA1s* mutation)^9,10^. Understanding how trisomy 21 leads to cell pathology will be important for development of traditional therapeutics for DS, and our results provide substantial new insights into this.

In addition, gene therapies are being developed for monogenic disorders due to the ongoing revolution in gene editing and in vivo delivery technologies^25^. Such hopeful progress, however, has not been relevant for chromosomal imbalances, involving hundreds of genes across a chromosome. Here we demonstrate that even without identification of pathogenic genes, insertion of a single epigenetic switch to suppress chromosome-wide transcription can effectively mitigate cell pathogenesis and normalize phenotypic outcome.

## Results

### A system to examine trisomy 21 effects in identical cell populations

**Fig. 1:**
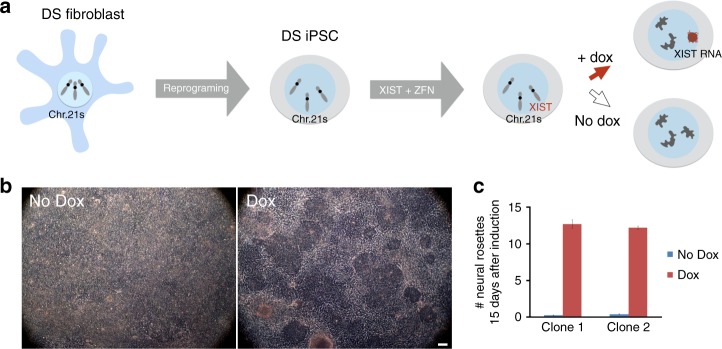
System to study the effect of trisomy 21 expression on DS-related pathologies. **a** Schematic of the inducible *XIST* RNA-mediated silencing system in Down syndrome iPSCs, in which *XIST* induces formation of a condensed, heterochromatic chromosome 21 “Barr Body”. **b** Neural stem cell (rosette) formation after 15 days neural differentiation, with and without induced expression to silence one chromosome 21. The samples treated with dox have significantly more neural stem cells at this time point. Scale bar: 100 μm. **c** Quantification of the number of neural rosettes at day 15 for two of the isogenic *XIST*-transgenic subclones. The iPS cells used were from a male, but because regulatory counting elements are removed from the *XIST* cDNA, this system is also compatible with female cells. Error bars represent s.e.m. from three independent experiments

Figure 1a summarizes the experimental design in which a doxycycline-inducible full-length *XIST* cDNA was inserted into one of three chromosome 21s in iPSCs (derived from a male DS patient) as previously described^6^. This prior study focused on showing that a full-length cDNA could be targeted into chromosome 21 and the *XIST* RNA properly localized to induce transcriptional silencing across that chromosome *in* *cis*, shown in undifferentiated iPSCs. Here we investigate whether trisomy silencing can normalize hematopoietic cell phenotypes using a previously characterized all-isogenic panel of DS iPSC subclones, including four independent *XIST*-transgenic clones, the non-transgenic parental trisomic line, and an isogenic disomic subclone (from a cell, which spontaneously lost one chromosome 21). The inducible system compares the effects of reducing trisomy 21 over-expression in parallel cultures of otherwise identical cell populations, thus minimizing sources of variation between even isogenic clones^26–28^.

Prior to considering effects in hematopoiesis, it is instructive to consider that expression of transgenic *XIST* clearly enhances proliferation of iPSCs and formation of neural progenitors (Fig. 1b and previously reported^6^). These findings affirm that *XIST* expression is not toxic, but instead can have beneficial effects on cell proliferation and viability. The enhanced kinetics of neural stem cell formation upon trisomy silencing may reflect correction of a developmental defect, however a deficit in neural stem cell formation is not an established phenotype of DS, and was not apparent in comparison of non-isogenic trisomic and disomic iPSCs (which showed general variability between lines)^29^. Therefore, it remained possible that trisomy silencing would enhance proliferation and production of any cell type, if this relieves a general aneuploidy stress. Hence, it was of interest to determine if *XIST* expression would accentuate, have no effect, or actually decrease (normalize) the well-established overproduction of hematopoietic cell types seen in DS children. If there are cell type-specific effects consistent with clinical impact, this would support that trisomy silencing corrects the developmental program, not just general stress.

### Trisomy 21 silencing normalizes production of CFU-Mk and CFU-E

**Fig. 2:**
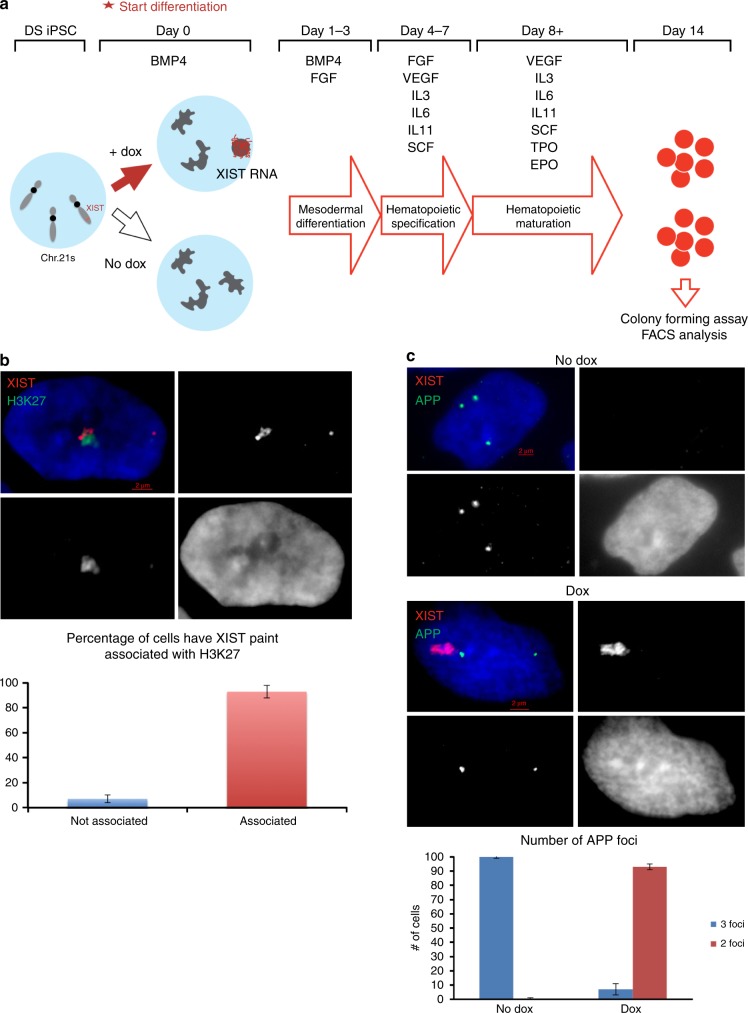
Trisomy silencing during hematopoietic differentiation. **a** Schematic of hematopoietic differentiation from DS iPSCs. **b** Association of H3K27me3 heterochromatic mark with *XIST* RNA in day 9 purified CD34^+^ differentiated cells treated with dox. Quantification shows over 90% of cells have H3K27me3 marker associated with XIST paints. **c** RNA FISH of *APP* and *XIST* on day 9 differentiating cells. *APP* RNA transcription foci from all three alleles in untreated cells (top). In treated cells (bottom), only two *APP* transcription foci are detected, indicating transcriptional silencing induced by *XIST* expression. Quantification shows over 90% of *XIST* expressing CD34^+^ cells have only two *APP* transcription foci (which are not from *XIST* expressing chromosome). Error bars for **b** and **c** represent s.e.m. from three independent scorings

The elevated risk for TMD and AMKL in DS individuals is characterized by markedly increased production of megakaryocytes and erythrocytes, as affirmed in DS fetal liver cells in vitro^9–11^ and in studies using DS iPSCs that mirror fetal hematopoiesis^12,20^, but not another that reflected primitive hematopoiesis, an earlier stage^30^. This is consistent with other evidence that hyper-proliferation arises during fetal hematopoiesis, explaining why risks of TMD and AMKL subside after a few years of age. Hence, we adopted a protocol that was shown to mimic fetal hematopoiesis from iPSCs differentiated through embryoid bodies (EB), using a defined cytokine cocktail as reported by Maclean et al.^20^ (Fig. 2a).

**Fig. 3:**
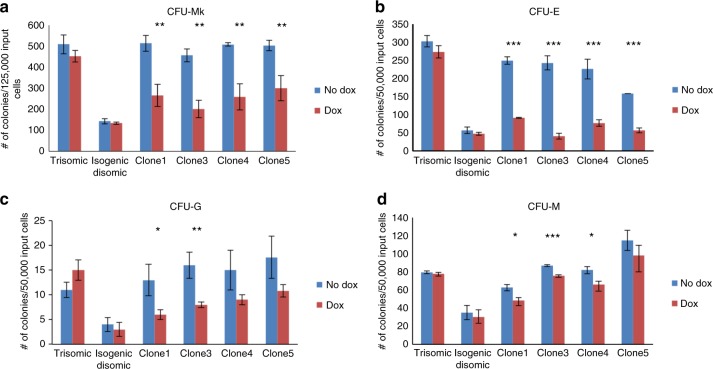
Effect of *XIST*-induced trisomy silencing on colony-forming potential of DS iPSCs. Colony-forming potential of multiple isogenic transgenic DS iPS lines with and without *XIST*-induced chromosome 21 silencing for **a** megakaryocytes, **b** erythrocytes, **c** granulocytes, and **d** monocytes. Experiments were repeated at least three times. Error bars represent s.e.m. from three independent experiments and *P* values were calculated by Student *t* test; *P < 0.05, **P < 0.01, ***P < 0.001

Our prior study demonstrated *XIST*-induced chromosome silencing is largely complete within 3–5 days of induction^6^, but we confirmed chromosome silencing in our hematopoietic differentiation system by examining *XIST* RNA localization, H3K27me3 (hallmark of heterochromatin), and silencing of the *APP* transcriptional foci from the *XIST*-coated chromosome. After 9 days of differentiation we disaggregated EB and sorted cells using the CD34 marker broadly used to enrich for the hematopoietic lineage^31^. More than 90% of *XIST*-positive CD34^+^ cells exhibited a well-localized *XIST* RNA accumulation and H3K27me3 across the chromosome (co-localized with *XIST* RNA) (Fig. 2b) and silencing of the *APP* transcription focus from the targeted chromosome (Fig. 2c). These experiments utilized parallel cultures (with and without dox-induced *XIST*) for four independent transgenic subclones (termed clones 1, 3, 4, and 5, which were previously characterized^6^), as well as the non-transgenic trisomic parental line and a disomic subclone (also plus/minus doxycycline). Comparison of the non-transgenic trisomic parental and disomic subclone is included for reference; although this is just one comparison of different subclones, our findings are highly consistent with other studies showing that DS trisomic iPSCs generate more megakaryocyte and erythrocyte colonies (Fig. 3a, b)^11,12,20^.

Our first goal was to address whether induced *XIST* expression can mimic results of normal euploid cells and rebalance overproduction of hematopoietic cell types, examined first for the differentiation end state by colony-forming assays. EB at day 14, which contain hematopoietic progenitor cells, were dissociated and equal cell numbers plated for colony-forming assay or fluorescence-activated cell sorting (FACS) analysis (Fig. 2a). The morphology of colonies is shown in Supplementary Figure 1.

The first point to note is that doxycycline treatment of the non-transgenic parental or disomic lines had no significant effect on the production of the four types of hematopoietic cell colonies, affirming that the effect of doxycycline on the transgenic clones is due to *XIST* expression to induce chromosome silencing. Most importantly, a marked, statistically significant reduction (50–80%) in megakaryocyte (CFU-Mk) and erythrocyte (CFU-E) colonies is consistently seen in parallel comparisons for all four clones (±*XIST*) (Fig. 3a, b). These observations of reduced CFU-MK and CFU-E after *XIST* expression are consistent with overproduction in DS newborns of erythrocytes and megakaryocytes, and mirror results from comparisons of hematopoiesis from trisomic DS versus normal iPSCs. Therefore, these results provide the first critical demonstration that cellular phenotype can be normalized or greatly mitigated by *XIST* RNA-mediated chromosome silencing.

While the expected effect of trisomy 21 on CFU-MK and CFU-E is greatest and firmly established, it is much less clear whether trisomy 21 affects production of granulocytes and monocytes^11,12,20,30^. The direct comparison of identical cell populations (±*XIST*) may provide an opportunity to detect any more subtle differences. Results suggest there is a modest but reproducible decrease in the reductions of granulocyte colonies (CFU-G) as a function of chromosome 21 silencing (Fig. 3c). The low numbers of CFU-G (in all samples) makes this comparison more difficult, but a statistically significant difference was present for some single pairwise comparisons and a trend of reduced CFU-G colonies evident for all pairwise comparisons of trisomic versus disomic state. Thus, these results suggest trisomy 21 enhances granulocyte production, but to a much lesser degree than megakaryocytes and erythrocytes. Data for monocyte colonies (CFU-M) showed very small differences, repeatedly in three replicate experiments of four clones, but overall results indicate monocyte colony production is largely unaffected.

These results demonstrate that *XIST*-mediated silencing of one chromosome 21 in DS patient-derived cells can normalize the clearly aberrant overproduction specifically of CFU-Mk and CFU-E in vitro. As further considered in Discussion, this alone is a major milestone as it shows proof of feasibility that a single gene insertion can correct pathogenesis of an established phenotype, one which confers high susceptibility to TMD and leukemia.

### Excess cells arise as CD43^+^ progenitors emerge from the endothelial-to-hematopoietic transition

**Fig. 4:**
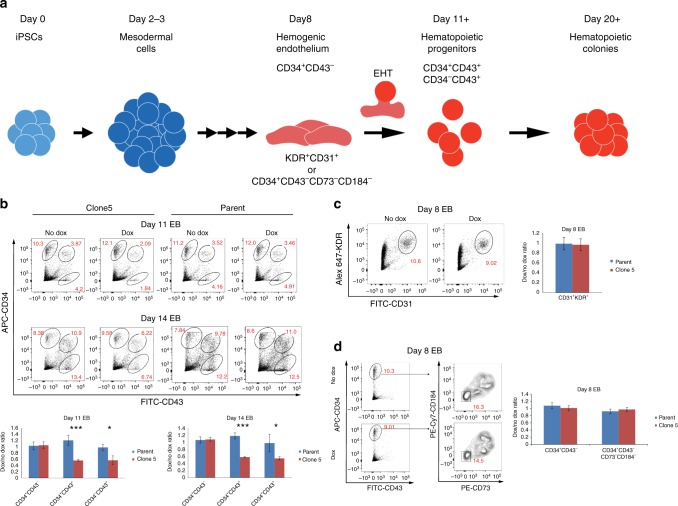
Impact of trisomy silencing on hematopoietic populations during differentiation. **a** A simplified schematic of steps in the hematopoietic differentiation process with markers used to define specific cell population. EHT: endothelial to hematopoietic transition. **b** Early hematopoietic progenitor populations detected at day 11 and 14 differentiation for clone 5. Quantifications are represented as the ratio of doxcycycline-treated cells to untreated cells. Transgenic cells, which *XIST* is induced have less CD43^+^ early hematopoietic progenitor cells at both day 11 and 14 of differentiation. **c**, **d** Hemogenic endothelium-like populations identified during hematopoietic differentiation for clone 5. Quantifications are represented as the ratio of treated cells to untreated cells. Chromosome 21 silencing does not affect the formation of hemogenic endothelium-like populations at day 8 of differentiation, using either of the two sets of markers to define this population. Error bars represent s.e.m. from five independent experiments and *P* values were calculated by Student *t* test; *P < 0.05, ***P < 0.001

Given that *XIST* expression successfully normalized the end product of hematopoiesis, we next examined which steps in hematopoiesis are most impacted by trisomy silencing. Fetal hematopoiesis is a complex process and can be modeled by in vitro differentiation of pluripotent stem cells^31–40^. Several major stages in hematopoiesis are outlined in Fig. 4a, along with markers used to isolate distinct populations.

The HE is a small subset of endothelial cells, which can produce hematopoietic stem cells (HSCs) during early embryogenesis. HE is difficult to identify as it only exists transiently and expresses both hematopoietic and endothelial markers (Fig. 4a)^41^. The formation of HSCs from HE is known as the endothelial-to-hematopoietic transition (EHT). HSCs are capable of self-renewal and differentiation to all hematopoietic lineages. Increased numbers of hematopoietic progenitor cells were demonstrated in studies using DS fetal livers^9,10^and iPSC systems^12,20,30^. Therefore, we next tested whether *XIST*-induced trisomy silencing would reduce production of hematopoietic progenitor cells.

Hematopoietic differentiation through EB was induced from *XIST*-transgenic DS iPSCs, with and without *XIST* induction, and different cell populations were examined at different times (Fig. 4). CD34 is widely used to identify cell populations containing hematopoietic stem and progenitor cells, whereas CD43 is the earliest marker of full hematopoietic commitment following EHT in vitro^40^. Hence, using both markers we identify three populations (CD34^+^/CD43^−^, CD34^+^/CD43^+^, and CD34^−^/CD43^+^). The two CD43^+^ populations are expanding while the earlier CD34^+^CD43^−^ population shrinks from day 11 to day 14. As CD43 is the earliest marker, the two CD43^+^ populations most closely represent committed hematopoietic progenitors.

As shown in Fig. 4b, induced chromosome silencing resulted in significant reduction in the two CD43^+^ but not the CD34^+^CD43^−^ population. Therefore, trisomy silencing again normalizes the cell phenotype, recapitulating the decrease in CD43^+^ cells previously shown for normal relative to trisomic cells. Hence overproduction of differentiated hematopoietic colonies (Fig. 3) is preceded by overproduction of hematopoietic progenitors, but it is not known whether excess progenitors initially arise at an earlier step. Given that trisomy 21 status strongly impacted the CD43^+^ progenitors but not the CD34^+^/CD43^−^ cells, we used this approach to ask if the developmentally earlier population of the HE is also overproduced in trisomic cells. Alternatively, if the HE population numbers are unaffected, this would indicate the excess of CD43^+^ committed hematopoietic progenitors first emerge following the EHT process. KDR^+^CD31^+^ populations at day 8 differentiation include HE-like cells^39^, and consistent with earlier observations, we found no significant difference in this population (Fig. 4c)^12,20^. A recent study demonstrated that HE is further enriched in the CD34^+^CD43^−^CD73^−^CD184^−^ population^31^. Analysis of this population demonstrates no significant difference (Fig. 4d). Thus, using two different sets of markers no effect was seen on the HE-like population. Collectively, results indicate trisomy 21 enhances the EHT process or steps closely thereafter, leading to overproduction of CD43^+^ progenitors. As considered in the Discussion, this with other findings suggest a potentially overactive EHT process.

### CFU-Mk and CFU-E production from CD43^+^ cells also increases

**Fig. 5:**
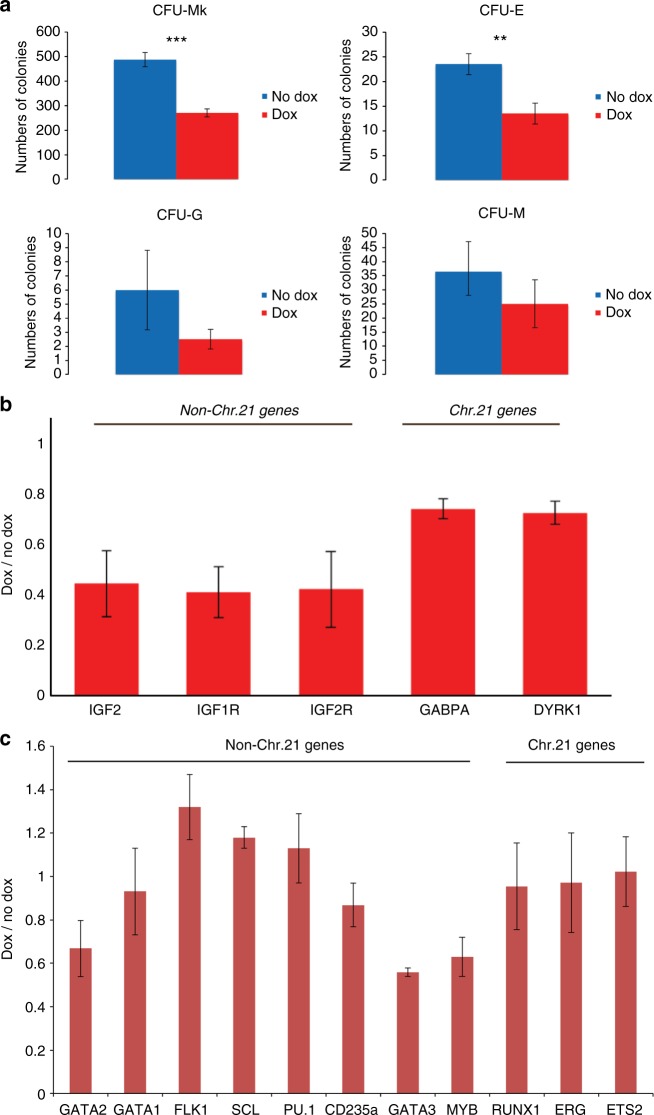
Effects of trisomy silencing on day 14 purified CD43^+^ hematopoietic progenitors. **a** Colony-forming potential from equal numbers of purified day 14 differentiated CD43^+^ hematopoietic progenitor cells for (i) megakaryocytes, (ii) erythrocytes, (iii) granulocytes, and (iv) monocytes. **b**, **c** Gene expression analysis on **b** three IGF signaling-related genes and two chromosome 21 genes in purified day 14 CD43^+^ hematopoietic progenitors, and on **c** a panel of hematopoietic regulators, which are dynamically changing in different hematopoietic subpopulations. Expression levels were normalized to *GAPDH* and represented as the ratio of treated cells to untreated cells. Error bars represent s.e.m. from three independent experiments and *P* values were calculated by Student *t* test; *P < 0.05, **P < 0.01, ***P < 0.001

Studies of DS fetal liver cells showed more hematopoietic colonies generated from equal numbers of progenitor cells^9–11^. This could reflect effects of trisomy on the in vivo niche, or an impact on the cell autonomous differentiation competence. We thus used our experimental system to determine if trisomy 21 expression affects formation of various colony types from equal numbers of CD43^+^ progenitors purified from day 14 EB. There was only a marginally significant difference in CFU-G, and slight reduction in CFU-M, but consistent with studies of fetal livers, results show marked decreases in CFU-Mk and CFU-E formation in chromosome 21 silenced samples of transgenic subclones, (Fig. 5a). In addition, the CFU-Mk colonies in treated samples were usually smaller than in untreated samples, reflecting enhanced megakaryocyte proliferation in DS. Therefore, trisomy 21 expression not only causes overproduction of CD43^+^ progenitors but also increases their inherent capacity to produce more megakaryocytes and erythrocytes. Importantly, results show the latter effect is due to cell intrinsic properties, likely through excess production of megakaryocyte-erythroid progenitors within the CD43^+^ hematopoietic progenitor population.

### Overactive IGF signaling implicated in overproduction of CD43^+^ cells

Finally, we explored the utility of this system to examine gene expression changes as a function of silencing the third chromosome 21. The heterogeneous nature of hematopoietic cell populations (even as defined by specific markers) and the rapidly changing levels of hematopoietic regulators during hematopoiesis likely contribute to observations in multiple studies that consistent changes in gene expression due to trisomy 21, even for known hematopoietic regulators encoded on chromosome 21, have alluded identification^11,20^. Since there is some expression variability (or noise) inherent in different stem cell clones, we tested whether inducible chromosome silencing might be able to discern consistent changes, focusing on a small panel of genes of interest, as previously examined by Maclean et al.^20^. This included several genes involved in IGF signaling, which were of particular interest.

The mechanism whereby trisomy 21 itself (without *GATA1s*) leads to overproduction of megakaryocytes and erythrocytes remains an important question, but as recently discussed, differences in the expression or responsiveness to the developmentally regulated IGF signaling pathway remain an attractive candidate^22^. The IGF pathway is well-established to broadly impact cell proliferation and is linked to a variety of other cancer types^42^, and was implicated as key to fetal hematopoiesis and DS-associated TMD and DS-AMKL^21^. Klusmann et al.^21^ showed that fetal hematopoiesis specifically is reliant on IGF signaling. They found IGF signaling genes substantially over-expressed in DS-AMKL compared to non-DS-AMKL, and that DS cells were especially sensitive to IGF signaling inhibition^21^. Given the importance of IGF signaling in other cancers, it would be significant if differences between trisomic and euploid hematopoietic cells could be corroborated, particularly in cells prior to cancer genesis. Since Klusmann et al. examined leukemic cells which had the *GATA1s* mutation, they could not address whether trisomy 21 alone promotes overactive IGF signaling.

We performed RT-qPCR (reverse transcription quantitative polymerase chain reaction) on RNA from purified day 14 CD43^+^ cells, examined in three independent experiments comparing parallel cultures with and without dox-induced *XIST*. For many genes, most of which are highly dynamic and cell type-specific hematopoietic regulators, no significant patterns were evident (Fig. 5a–c), similar to findings of Maclean et al.^20^. In contrast to Maclean et al., however, we did detect consistent, statistically significant differences in five of these same genes, all of which have been discussed as potentially contributing to trisomy 21 hematopathology (Fig. 5b). For chromosome 21 genes *DYRK1A* and *GABPA*, a reduction in mRNA levels close to the 1/3 level expected (due to silencing one of the three chromosome 21s) was evident (Fig. 5b). The sensitivity of our system to detect such modest changes likely also reflects wide expression of these two broad regulators in much of the heterogeneous CD43^+^ hematopoietic cell population; we note that *GABPA* and *DYRK1A* are expressed early in undifferentiated iPSCs (Jiang et al.^6^ and unpublished). Most importantly, results demonstrate that indeed there is increased expression of IGF signaling genes (not on chromosome 21) in the trisomic versus trisomy silenced state (Fig. 5b). All three *IGF* signaling genes examined, *IGF2*, *IGF1R*, and *IGF2R*, were markedly downregulated by about 60% in trisomy silenced CD43^+^ cells.

**Fig. 6:**
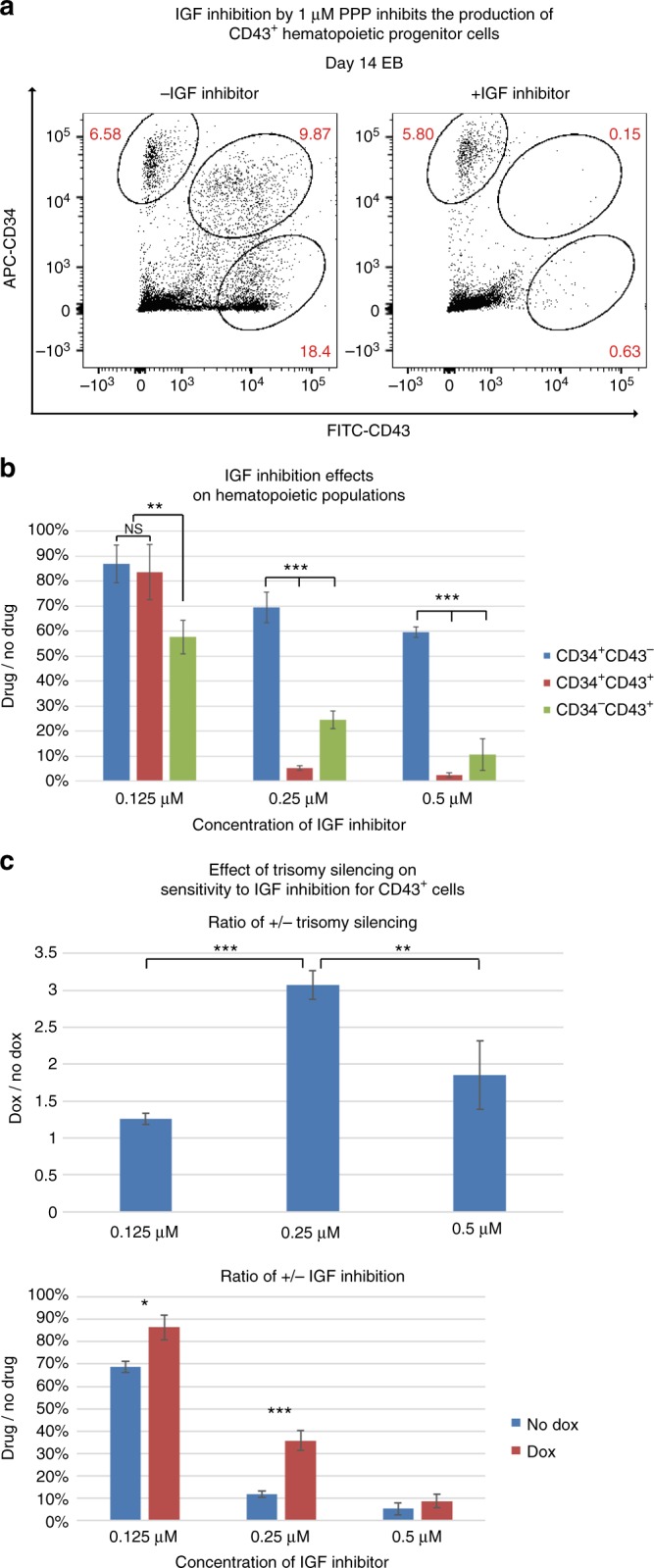
Trisomy 21 contributes to increased sensitivity of CD43^+^ hematopoietic progenitor production to IGF inhibition. **a** Inhibition of IGF signaling by 1 μM PPP, an IGF inhibitor, has distinct effects on the CD34^+^ and CD43^+^ populations. Production of CD43^+^ early hematopoietic progenitor populations are more sensitive to IGF inhibition, whereas CD34^+^CD43^−^ hemogenic endothelium-enriched population is only slightly affected. **b** Analysis of the effects of IGF inhibition at three lower concentrations identifies the window of sensitivity for the CD43^+^ cells. Drug/no drug ratios were calculated by the percentage of each population in treated cultures divided by the percentage of each population in untreated cultures. **c** Trisomy silencing reduces the sensitivity of CD43^+^ cell production to IGF signaling inhibition, suggesting greater reliance of trisomic CD43^+^ progenitors on IGF signaling. Drug/no drug ratios were calculated as in **b** and dox/no dox ratios were calculated by the drug/no drug ratio of dox-treated samples divided by drug/no drug ratio of samples without dox, to reflect the increased reliance on IGF signaling in trisomic CD43^+^ hematopoietic progenitors. Error bars represent s.e.m. from three independent experiments and *P* values were calculated by Student t test; **P < 0.01, ***P < 0.001

Next we used the same inhibitor (PPP) as Klusman et al.^21^ to examine effects on the three flow-sorted hematopoietic populations studied above (Fig. 6). Treatment began on day 8 and cells were evaluated on day 14 of differentiation. The first experiment using the same PPP concentration as Klusman et al. revealed that production specifically of the CD43^+^ progenitors was almost completely eliminated (Fig. 6a), whereas even this high concentration had very little effect on the earlier CD34^+^/CD43^−^ cells. Importantly, this showed that the drug was not generally toxic to cell proliferation, and affirms IGF signaling becomes important at this specific point in hematopoiesis (during or after the EHT process) (Fig. 6a).

Since IGF signaling was required for fetal hematopoiesis for both normal and trisomic samples, any greater effect of IGF signaling on trisomy 21 cell proliferation would only be evident within a certain concentration window of IGF inhibitor, as indicated by Klusman et al.^21^. Therefore, we first tested three lower inhibitor concentrations on trisomic cultures, which again affirmed greater effects on the CD43^+^ populations (Fig. 6b). Finally, results indicated substantially reduced sensitivity to IGF inhibition in the production of CD43^+^ cells when parallel cultures are induced to silence one chromosome 21 (Fig. 6c).

**Fig. 7:**
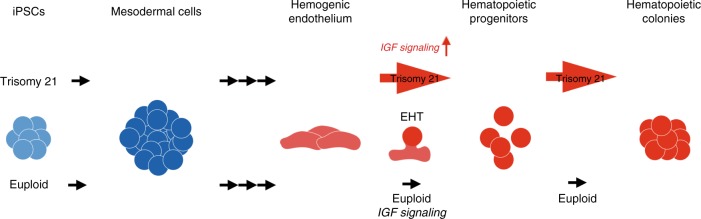
Schematic of how trisomy 21 affects distinct steps in hematopoiesis. Trisomy 21 initially increases the production of hematopoietic progenitor cells as they emerge from the endothelial-to-hematopoietic transition. A likely enhanced EHT process is accompanied by increased IGF signaling. Trisomy 21 also increases the colony-forming potential of these hematopoietic progenitor cells

Therefore, both gene expression analysis and the IGF inhibitor studies support the conclusion that trisomy 21 itself, prior to *GATA1s* mutation, increases IGF signaling, which in turn promotes excessive production of CD43^+^ hematopoietic progenitor cells in DS (Fig. 7). Results further demonstrate that expression of one gene, *XIST*, can sufficiently rebalance chromosome 21 expression to reduce excess IGF signaling and normalize pathological overproduction of hematopoietic cell populations. Figure 7 provides a summary of findings and model regarding the effect of trisomy 21 on distinct steps in the hematopoietic process.

## Discussion

Having demonstrated that an inserted *XIST* cDNA could repress transcription across one chromosome 21 in trisomic iPSCs^6^, a critical next question was whether trisomy silencing is sufficiently effective to normalize cell physiology and mitigate pathogenesis of known DS cellular phenotype(s)? Here we demonstrate that an *XIST* transgene can correct the complex regulatory mechanisms that underlie the established hematopoietic cell pathologies of DS. Trisomy silencing achieved by *XIST*-induced expression from one chromosome 21 sharply reduced the overproduction of megakaryocyte and erythrocyte colonies in multiple transgenic clones consistently. Similarly, this also normalized an excess of CD43^+^ hematopoietic progenitors but did not impact earlier CD34^+^/CD43^−^ population. Importantly, induced *XIST* expression in these same cell lines does not suppress proliferation of either iPSCs or neural stem cells, showing cell type specificity and supporting correction of the hematopoietic developmental program. Further investigation is required to determine if trisomy 21 causes a developmental delay in regulatory programs of early neurogenesis, however we note that the contrasting effects of trisomy 21 silencing on hematopoietic and neural cells is consistent with the different impact generally on the hematopoietic and cognitive systems in DS. Overall, results strongly support that *XIST* normalized the hematopoietic process by rebalancing dosage-sensitive chromosome 21 genes. This is promising for the prospect that developmental pathogenesis for other cell types could be normalized by *XIST*-induced trisomy silencing.

For several results studied here, we refer to findings of prior studies comparing trisomic versus normal cells^11,12,20,30^ to validate that the consequences (benefits) of chromosome 21 silencing on hematopoiesis approximates the effects of simply not having the extra chromosome 21. But on multiple points, findings also advance what is known and provide evidence for certain important hypotheses. Our results affirm that trisomic CD43^+^ hematopoietic progenitors (and differentiated cells) are produced in excess, however we also showed there was no effect on the number of the earlier HE-enriched population, identified by two sets of markers. This suggests the hyper-proliferative defect arises at or near the step known as the EHT, which produces CD43^+^ progenitors from the HE population. An enhanced EHT process also fits with the fact that the chromosome 21 *RUNX1* gene becomes expressed at this time and is required for production of committed hematopoietic progenitors from HE^12,43,44^. While other chromosome 21 genes may contribute, we postulate that trisomy for *RUNX1* likely drives an enhanced EHT process, although CD43^+^ cells may also have proliferative or survival advantage in the brief post-EHT interval prior to our assay.

One prior study had reported IGF signaling is overactive in leukemic cells for DS versus euploid patients, and is critical for fetal hematopoiesis^21^. Therefore, we examined and found increased expression of IGF signaling-related non-chromosome 21 genes in trisomic hematopoietic cells. We also showed that production of CD43^+^ progenitors (but not HE cells) is highly sensitive to IGF signaling inhibition, and the effect is greater in trisomic cells before chromosome silencing. This provides needed corroboration for the role of IGF signaling in fetal hematopoiesis and the important hypothesis of enhanced IGF signaling in DS fetal hematopoiesis. Notably, IGF signaling becomes important at the same step we found most impacted by trisomy 21, the formation of CD43^+^ progenitors, a step at which *RUNX1* is strongly implicated^12,43,44^, suggesting a potential link between *RUNX1* dosage and IGF signaling. In any case, our results demonstrate that the IGF-related effects of trisomy 21 are evident in non-leukemic hematopoietic progenitor cells (prior to GATA1s mutation). Our results do not rule out that GATA1s mutations also contribute as Klusmann et al.^21^ suggested, but these authors also acknowledged that trisomy 21 itself might enhance IGF signaling. We suggest that trisomy 21 and GATA1s could cooperate to push the same proliferation-promoting pathway to dangerous levels, potentially explaining why neither trisomy 21 nor GATA1s alone leads to TMD or leukemia. Given these results in the hematopoietic system, future studies should consider whether effects on IGF signaling might contribute to other aspects of the syndrome, such as the almost universal metabolic changes that make many individuals with trisomy 21 more prone to obesity or diabetes.

In addition to the broader significance of showing chromosome silencing can normalize pathogenesis of a DS cell phenotype, results here have direct relevance to common hematopoietic abnormalities in DS. Many children with DS develop clinically identified TMD; in most this will resolve naturally but TMD alone can bring substantial morbidity^22^, and this condition pre-identifies DS children at particularly high risk to acquire DS-AMKL. Although children with DS-AMKL generally have higher survival rates after chemotherapy than non-DS children, toxicity is also often even greater for DS children and the disease is still life-threatening^22^. Many groups are making progress on developing human HSCs from iPSCs for therapeutic purposes^45^. Additionally, bone marrow transplantation of genetically modified HSC is actively pursued for clinical applications and cord blood could serve as a more accessible source of HSCs for all DS newborns. Since it has been shown in mice that *Xist* can initiate chromosome silencing specifically in somatic hematopoietic progenitor cells^46^, it can now be considered that dosage-compensation of chromosome 21 expression in DS-TMD children might eventually be developed as a therapeutic ex vivo or in vivo strategy, or even conceivably in fetal liver in utero^47,48^ (the origin of cells that give rise to TMD and leukemia). It is also important to note that the blood system has relevance to other bodily systems impacted by trisomy 21. For example, the lymphoid system is impacted, causing ~20-fold increased risk for ALL^49^, a more common childhood leukemia for which success of chemotherapy is substantially less than for DS-AMKL. While we focus here on myeloid differentiation, the hematopoietic progenitors studied here (and corrected by trisomy silencing) can also give rise to cells of the lymphoid system, which could also be studied. Any ability to normalize the transcriptome might also mitigate immune defects, such as high viral susceptibility, inflammation, and autoimmune defects^16–18,50^. Furthermore, many recent studies suggest a link between immune system, inflammation, and neurodegenerative diseases, such as the Alzheimer disease prevalent in DS.

Understanding the effects of trisomy 21 on different cell types and pathways will be important for identification of potential drug targets (e.g. IGF signaling). However, any possibility of chromosome therapy would bring the advantage that silencing genes across one chromosome 21 would circumvent difficulty of unraveling the complex biology arising from triplication of close to 300 genes. The first step in development of any gene therapy approach is to demonstrate that the genetic abnormality can be corrected in vitro. Since *XIST*-mediated chromosome silencing is not traditional correction of a genetic mutation, it was essential to show that this could normalize a DS cell phenotype and developmental program. Importantly, we show that correction of a known DS cellular phenotype can be achieved even without knowing which or how many specific genes contribute to these abnormalities. It will be important to test the efficacy of *XIST* and the potential of this approach for other dysregulated systems in DS. While several challenges remain, these positive findings have implications for the potential future development of *XIST* transgenes for chromosome therapy, for various aspects of DS, and potentially other chromosomal disorders. Given results shown here and the ongoing revolution in gene-editing technology^51^, the transformative potential that insertion of a single gene could mitigate some effects of trisomy 21 to improve the lives of people with DS, and potentially other dosage imbalances, merits further testing and development.

To summarize, this study demonstrates that transcriptional repression of chromosome 21 induced by a single *XIST* transgene is sufficiently effective in normalizing cell physiology that it mitigates a major developmental pathology in DS. Hence, this inducible chromosome silencing provides a valuable experimental approach to determine the most direct effects of trisomy 21 on cell function and development, important for traditional drug therapeutics. In addition, results demonstrate success at an important milestone, one that in our view was a pre-requisite for any prospect that a targeted *XIST* transgene could potentially become the basis of a therapeutic strategy. While other milestones remain, these results encourage further exploration and development of this transformative concept.

## Methods

### Inducible *XIST*-mediated chromosome silencing system

The inducible *XIST*-mediated chromosome silencing system was built on DS iPSC parental line (DS1-iPS4) provided by George Q. Daley^6^. Briefly, doxycycline-controlled *XIST* transgene was inserted into *DYRK1A* locus on chromosome 21 and a transgene carrying the doxycycline control element (rtTA) was inserted into *AAVS1* locus on chromosome 19 by electroporation. Isogenic disomic clone that spontaneously lost one chromosome 21 after electroporation was isolated and served as a control for expression of the dox control element in the disomic ± dox comparison in Fig. 3.

### iPSC culture

iPSCs were maintained on irradiated mouse embryonic fibroblasts (R&D Systems) in iPSC medium containing KnockOut-DMEM/F12 supplemented with 20% KnockOut serum replacement (ThermoFisher), 1× GlutaMax (ThermoFisher), 100 μM non-essential amino acids (ThermoFisher), 100 μM β-mercaptoethanol (Sigma), and 10 ng/ml fibroblast growth factor (FGF)-β (ThermoFisher). Cultures were passaged with 1 mg/ml collagenase type IV (ThermoFisher) every week.

### Neural differentiation

The differentiation and analysis of neural stem cells was previously described^6^. Briefly, iPSCs were dissociated with Accutase (Innovative Cell Technologies) and 4 × 10^5^ cells were plated on Matrigel in mTeSR1 medium (Stemcell technologies). Neural differentiation started when cell cultures reached 90–100% confluence by neural induction medium, a 1:1 mixture of N2- and B27-containing media supplemented with 500 ng/ml Noggin (R&D Systems), 10 μM SB431542 (Tocris Bioscience), and 1 μM retinoic acid (Sigma, cat#: R2625). The numbers of neural rosettes were counted at day 15.

### RNA fluorescence in situ hybridization and immunostaining

Immunostaining for H3K27me3 (Millipore, 07–449) and RNA fluorescence in situ hybrization (FISH) for *XIST* and *APP* were performed according to our previous published protocol^6,52^. Briefly, cells cultured on coverslips were extracted with triton (Roche) for 3 min and fixed in 4% paraformaldehyde in phosphate-buffered saline (PBS) for 10 min. Cells were then dehydrated in 100% cold ethanol for 10 min and air-dried prior to hybridization. The *XIST* probe was a 14 kb *XIST* cDNA in pGEM-7Zf(+) (Promega) and the APP probe is a BAC from BACPAC Resourses (RP11-910G8). DNA probes were labeled by nick translation with either biotin-11-dUTP or digoxigenin-16-dUTP (Roche). Fifty nanograms of labeled probes, with cot-1 competitor, was resuspended in 100% formaldehyde, followed by denaturation in 80 °C for 10 min. Hybridization was performed in 1:1 mixture of denatured probes and 50% formamide hybridization buffer (as described^52^) supplemented with 2 U/μl of RNasin Plus RNase inhibitor (Promega) for 3 h or overnight at 37 °C. Cells were then washed three times for 20 min each, followed by detection with anti-dig antibodies or fluorescein-avidin and DNA stained with DAPI. For immunostaining with RNA FISH, cells were immunostained first and fixed in 4% paraformaldehyde before RNA FISH. All antibodies were diluted at 1:500 ratio.

### Hematopoietic differentiation

The base medium for hematopoietic differentiation consisted of 50 μg/ml of Ascorbic Acid (Sigma A4544), 150 μg/ml of Transferrin (Roche), and 4 × 10^−4^ M of Monothioglycerol (Sigma M6145) in StemPro-34 medium (Invitrogen). iPSCs were passaged from feeders to growth factor reduced matrigel (Corning #356230) for 24–48 h to remove feeder cells. At day 0, iPSCs were lifted and broken into small pieces by collagenase B (Roche) then cultured in base medium supplemented with 10 ng/ml of BMP4 in 5% oxygen environment. Cells were cultured in suspension in low attachment plate (Corning) to facilitate formation of EB. *XIST* expression was induced by doxycycline treatment (500 ng/ml) at day 0. At day 1, EBs were cultured in base medium supplemented with 10 ng/ml of BMP4 and 3 ng/ml of FGF to induce hematopoietic differentiation. At day 4, EBs were transferred to base medium supplemented with 10 ng/ml of VEGF, 1 ng/ml of FGF, 40 ng/ml of IL-3, 10 ng/ml of IL-6, 5 ng/ml of IL-11, and 100 ng/ml of SCF. Starting from day 8, FGF was replaced by 50 ng of TPO and 4U/ml of EPO in the cytokine cocktail^20^. After 14 days of differentiation, EBs were dissociated and single cells were plated in MegaCult medium for generating megakaryocytic colonies and MethoCult medium for erythroid, monocyte, and granulocyte colonies (Stem Cell Technologies). After 10 days, colonies were fixed and scored according to the manufacturer’s instructions. All cytokines were purchased from Pepro Tech except Erythropoietin (R&D Systems).

### Flow cytometry

Cells from dissociated EB were filtered through 50 μm filter (Partec) before stained for FACS analysis. All antibodies were purchased from BD Bioscience, including anti-CD34-APC (#555824), anti-CD43^−^FITC (#555475), anti-CD31-PE (#555446), anti-CD31-FITC (#555445), anti-CD73-PE (#550257), anti-CD184- PE-Cy7 (#560669), and anti-CD309- Alexa Flour 647 (#560495). Cells were stained for 30 minutes at 4 degree in the dark then washed twice with PBS supplemented with 2% FBS before flow cytometry. DAPI was used for selection of live cells. All antibodies for flow cytometry were diluted at 1:5 ratio.

### RT-qPCR

RNA was isolated from purified CD43^+^ hematopoietic progenitor cells using RNeasy kit (Qiagen). cDNA was generated by iScript cDNA kit (BioRad). IQ SYBR Green supermix (BioRad) was used for quantitative PCR (qPCR) reactions. All reactions were done in triplicate and the expression levels were normalized by expression of GAPDH.

### IGF signaling inhibition by PPP

Hematopoietic differentiation cultures were treated with various concentrations of PPP, an IGF1R inhibitor (Millipore), to inhibit IGF signaling as described in Klusmann et al.^21^. Briefly, differentiating EB were treated with PPP starting from day 8, the time HE forms, to day 14, the time we assayed the amount of different hematopoietic populations. The effect of PPP on each population was calculated by the percentage of that population in treated cultures divided by the percentage of that population in untreated cultures.

### Statistical analysis

All experiments were done at least in triplicate and repeated independently. For cell counting, at least three random regions on the slides were scored for 100 cells for each experiment. For colony-forming assays, three independent plates (for MethoCult) and three independent slides (for MegaCult) were scored for the number of colonies of each type. For flow cytometry analysis, five independent experiments were performed. For qPCR, reactions were performed in triplicate in three independent experiments. One-tailed Student *t* test was used to determine the significant level of differences between treated and untreated samples. Differences were considered to be significant when *P* < 0.05. Error bars represent standard error of the mean.

## Electronic supplementary material

Supplementary Information

Reporting Summary

## Data Availability

The datasets generated during and/or analyzed during the current study are available from the corresponding author on reasonable request.
