# Supplementary material for: Trisomy silencing by XIST normalizes Down syndrome cell pathogenesis demonstrated for hematopoietic defects in vitro
Source: Nat Commun. 2018 Dec 5;9:5180. doi: 10.1038/s41467-018-07630-y (PMC6281598; doi:10.1038/s41467-018-07630-y)
Supplement: Supplementary file 1 — Supplementary Information [file 41467_2018_7630_MOESM1_ESM.pdf]

SUPPLEMENTARY INFORMATION

Trisomy Silencing by XIST normalizes Down Syndrome cell pathogenesis demonstrated for hematopoietic defects *in vitro*

Chiang et. al

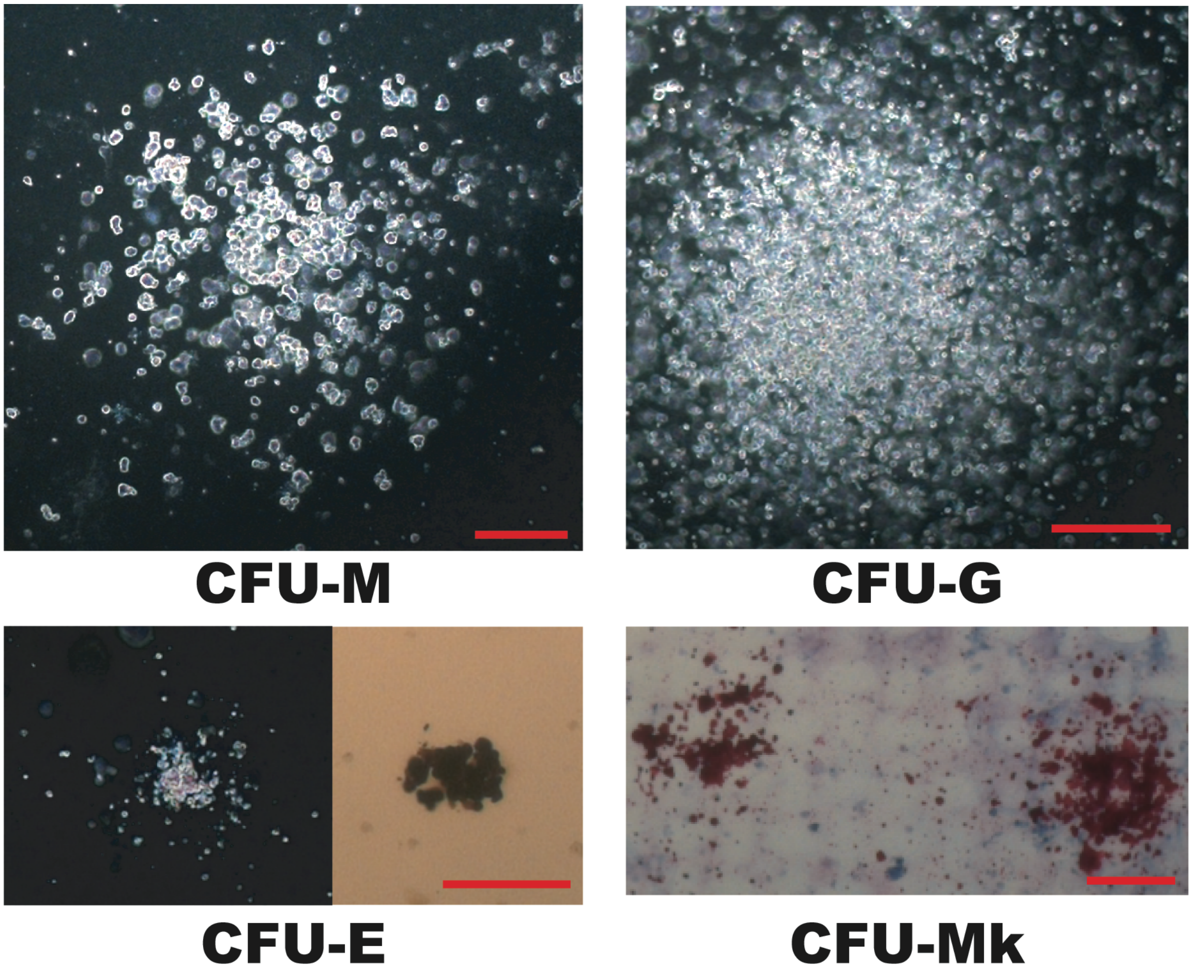

Figure S1 Morphology of hematopoietic colonies.

Morphologies of CFU-M, CFU-G, CFU-E, and CFU-Mk are shown here. For CFU-E, the left and right fields show before and after DAB staining, respectively.
CFU-M: Monocyte; CFU-G: Granulocytes; CFU-E: Erythrocytes; CFU-Mk: Megakaryocytes.
