# Supplementary material for: Trisomy silencing by XIST normalizes Down syndrome cell pathogenesis demonstrated for hematopoietic defects in vitro
Source: Nat Commun. 2018 Dec 5;9:5180. doi: 10.1038/s41467-018-07630-y (PMC6281598; doi:10.1038/s41467-018-07630-y)
Supplement: Supplementary file 2 — Reporting Summary [file 41467_2018_7630_MOESM2_ESM.pdf]

nature research

Corresponding author(s): Jeanne Lawrence

Reporting Summary

Nature Research wishes to improve the reproducibility of the work that we publish. This form provides structure for consistency and transparency in reporting. For further information on Nature Research policies, see Authors & Referees and the Editorial Policy Checklist.

Statistical parameters

When statistical analyses are reported, confirm that the following items are present in the relevant location (e.g. figure legend, table legend, main text, or Methods section).

n/a	Confirmed	
☐	☒	The exact sample size (n) for each experimental group/condition, given as a discrete number and unit of measurement
☐	☒	An indication of whether measurements were taken from distinct samples or whether the same sample was measured repeatedly
☐	☒	The statistical test(s) used AND whether they are one- or two-sided<br>*Only common tests should be described solely by name; describe more complex techniques in the Methods section.*
☐	☒	A description of all covariates tested
☐	☒	A description of any assumptions or corrections, such as tests of normality and adjustment for multiple comparisons
☐	☒	A full description of the statistics including central tendency (e.g. means) or other basic estimates (e.g. regression coefficient) AND variation (e.g. standard deviation) or associated estimates of uncertainty (e.g. confidence intervals)
☐	☒	For null hypothesis testing, the test statistic (e.g. F, t, r) with confidence intervals, effect sizes, degrees of freedom and P value noted<br>*Give P values as exact values whenever suitable.*
☐	☒	For Bayesian analysis, information on the choice of priors and Markov chain Monte Carlo settings
☐	☒	For hierarchical and complex designs, identification of the appropriate level for tests and full reporting of outcomes
☐	☒	Estimates of effect sizes (e.g. Cohen's d, Pearson's r), indicating how they were calculated
☐	☒	Clearly defined error bars<br>*State explicitly what error bars represent (e.g. SD, SE, CI)*

Our web collection on statistics for biologists may be useful.

Software and code

Policy information about availability of computer code

Data collection	There was no code used in this study
Data analysis	There was no code used in this study

For manuscripts utilizing custom algorithms or software that are central to the research but not yet described in published literature, software must be made available to editors/reviewers upon request. We strongly encourage code deposition in a community repository (e.g. GitHub). See the Nature Research guidelines for submitting code & software for further information.

Data

Policy information about availability of data

All manuscripts must include a data availability statement. This statement should provide the following information, where applicable:

- Accession codes, unique identifiers, or web links for publicly available datasets
- A list of figures that have associated raw data
- A description of any restrictions on data availability

The datasets generated during and/or analysed during the current study are available from the corresponding author on reasonable request.

Field-specific reporting

Please select the best fit for your research. If you are not sure, read the appropriate sections before making your selection.

☒ Life sciences ☐ Behavioural & social sciences ☐ Ecological, evolutionary & environmental sciences

For a reference copy of the document with all sections, see nature.com/authors/policies/ReportingSummary-flat.pdf

Life sciences study design

All studies must disclose on these points even when the disclosure is negative.

Sample size	There was no sample size calculation. We typically do experiments in triplicate and repeated at least three times independently to ensure the reproducibility.
Data exclusions	No data was excluded
Replication	All experiments were successfully repeated st least 3 times.
Randomization	This is not relevant to this study since this study specifically focus on comparisons between trisomic and trisomic silenced samples.
Blinding	the investigators were blinded to group allocation during data collection

Reporting for specific materials, systems and methods

Materials & experimental systems

n/a	Involved in the study
☐	☒ Unique biological materials
☐	☒ Antibodies
☐	☒ Eukaryotic cell lines
☒	☐ Palaeontology
☒	☐ Animals and other organisms
☒	☐ Human research participants

Methods

n/a	Involved in the study
☒	☐ ChIP-seq
☐	☒ Flow cytometry
☒	☐ MRI-based neuroimaging

Unique biological materials

Policy information about availability of materials

Obtaining unique materials	This study used XIST transgenic DS iPS cell line that we produced several years ago.

Antibodies

Antibodies used	anti-H3K27me3 (Millipore, 07–449) , anti-CD34-APC (BD#555824), anti-CD43-FITC (BD#555475), anti-CD31-PE (BD#555446), anti-CD31-FITC (BD#555445), anti-CD73-PE (BD#550257), anti-CD184- PE-Cy7 (BD#560669), and anti-CD309- Alexa Flour 647 (BD#560495).
Validation	Anti-trimethyl-Histone H3 (Lys27), also known as Anti-H3K27me3, is a highly published Rabbit Polyclonal Antibody. This protein A purified antibody is dot blot tested for trimethylated lysine 27 specificity and validated in WB, ICC, IP.<br>All BD anti-bodies are validated for FACS application.

Eukaryotic cell lines

Policy information about cell lines

Cell line source(s)	All cell lines used in this study were generated by our lab based on DS1-iPS4 line provided by George Q. Daley
Authentication	We requested and acquired DS1-iPS4 line from Daley lab

Mycoplasma contamination	all cell lines tested negative for mycoplasma contamination
Commonly misidentified lines (See ICLAC register)	*Name any commonly misidentified cell lines used in the study and provide a rationale for their use.*

Flow Cytometry

Plots

Confirm that:

- [x] The axis labels state the marker and fluorochrome used (e.g. CD4-FITC).
- [x] The axis scales are clearly visible. Include numbers along axes only for bottom left plot of group (a 'group' is an analysis of identical markers).
- [x] All plots are contour plots with outliers or pseudocolor plots.
- [x] A numerical value for number of cells or percentage (with statistics) is provided.

Methodology

Sample preparation	Embryoid bodies were dissociated with collagenase and trypsin to acquire single cells. Cells then washed with FACS buffer (PBS + 2% FBS) before stained with antibodies (80 ul FACS buffer + 20ul antibodies at 4 degree in dark for 30 minutes). After washed twice, cells were stained with DAPI before analysis.
Instrument	BD LSR II
Software	FlowJo
Cell population abundance	The purified CD34+ population are typically over 90% of the post-sort population
Gating strategy	the boundary between positive and negative population are defined based on the negative control and samples stained with single fluorescein

- [ ] Tick this box to confirm that a figure exemplifying the gating strategy is provided in the Supplementary Information.
